# Solitary Fibrous Tumors of Chest: Another Look with the Oncologic Perspective

**DOI:** 10.4274/balkanmedj.2017.0350

**Published:** 2017-05-15

**Authors:** Mert Saynak, Nirmal K. Veeramachaneni, Jessica L. Hubbs, Dilruba Okumuş, Lawrence B. Marks

**Affiliations:** 1 Department of Radiation Oncology, Trakya University School of Medicine, Edirne, Turkey; 2 Department of Cardiovascular Surgery, University of Kansas Medical Center, Kansas City, USA; 3 Department of Obstetrics and Gynecology, University of North Carolina, North Carolina, USA; 4 Department of Radiation Oncology, University of North Carolina, North Carolina, USA

**Keywords:** Solitary fibrous tumor, hemangiopericytoma, Sarcoma, Radiotherapy, Chemotherapy, molecular targeted therapies

## Abstract

Solitary ﬁbrous tumors are mesenchymal lesions that arise at a variety of sites, most commonly the pleura. Most patients are asymptomatic at diagnosis, with lesions being detected incidentally. Nevertheless, some patients present due to symptoms from local tumor compression (eg. of the airways and pulmonary parenchyma). Furthermore, radiological methods are not always conclusive in making a diagnosis, and thus, pathological analysis is often required. In the past three decades, immunohistochemical techniques have provided a gold standard in solitary ﬁbrous tumor diagnosis. The signature marker of solitary ﬁbrous tumor is the presence of the NAB2-STAT6 fusion that can be reliably detected with a STAT6 antibody. While solitary ﬁbrous tumors are most often benign, they can be malignant in 10-20% of the cases. Unfortunately, histological parameters are not always predictive of benign vs malignant solitary ﬁbrous tumors. As solitary ﬁbrous tumors are generally regarded as relatively chemoresistant tumors; treatment is often limited to localized treatment modalities. The optimal treatment of solitary ﬁbrous tumors appears to be complete surgical resection for both primary and local recurrent disease. However, in cases of suboptimal resection, large disease burden, or advanced recurrence, a multidisciplinary approach may be preferable. Specifically, radiotherapy for inoperable local disease can provide palliation/shrinkage. Given their sometimes -unpredictable and often- protracted clinical course, long-term follow-up post-resection is recommended.

## SOLITARY FIBROUS TUMORS OF CHEST: ANOTHER LOOK WITH THE ONCOLOGIC PERSPECTIVE

Solitary fibrous tumors (SFT) occur equally amongst men and women, and most often occur during middle age, although it is occasionally seen in younger people. About 70% of SFTs originate from thoracic structures, most often pleura (as was the case in the first-reported series described by Wagner in 1870) (1). SFTs have subsequently been described at almost every extrapleural anatomical site (2,3,4). The etiology of SFTs remains unknown, and no association has been demonstrated with environmental factors such as tobacco consumption or asbestos exposure (2).

Pleural SFTs arise most frequently from the visceral (vs. the parietal or mediastinal) pleura. Pathological characteristics of SFTs of the pleura first described by Klemperer and Robin (5) in 1931. Most tumors are slowly growing and are diagnosed incidentally as painless masses. However, although large tumors may cause local symptoms (e.g. pain and/or airway compression). Pathologically, SFTs can be either benign or malignant tumors (incidence ratio 7:1); however differentiation may be in conclusive (2,6).

Initial management of SFTs is largely surgical resection. The role of adjuvant treatment, or treatment for unresectable/recurrent disease, remains unclear and controversial, and warrants further investigation. We herein review the available English language literature and discuss the epidemiology, presentation, diagnosis, and treatment management of the malignant SFTs arising in the chest.

## PATHOLOGY

SFT microscopically tend to appear with hypocellular collagen rich areas alternating with a proliferation of uniform elongated spindled cells in a distribution causally. SFTs generally have positive staining for vimentin, CD34, and cytokeratin-negative. In addition, CD99 and bcl-2 are positive in ≈50% of SFTs. Also, SFTs are generally negative for S-100, carcinoembryonic antigen, and smooth muscle actin (7). However, the pathologic findings are somewhat variable and classification is thus imperfect.

A small percentage of SFTs possesses atypical features (8,9,10). Histological criteria to classify a SFT of the pleura as benign or malignant were described by England et al. (10) in 1989. These criteria include more than 4 mitoses per 10 high power ﬁelds, presence of necrosis, hypercellularity with crowding and overlapping of nuclei, and nuclear atypia. A tumor may have benign or malignant histologic features, but these features are not always predictive of the clinical behavior of the tumor (8,9,10).

The distinction between diffuse malignant mesothelioma (DMM) and SFT is usually not difficult because most of the latter are histologically benign. However, the distinction between DMM and malignant SFT can be challenging. In this setting, the gross appearance can be helpful because DMMs typically cause diffuse pleural thickening, and SFTs are usually solitary/localized masses, even when malignant. In addition, DMMs are positive for cytokeratin stains while SFTs are typically negative (although multifocal cytokeratin expression can be rarely occur). STAT6 is most often diagnostic of SFTs (11). In the light of recent developments, nuclear staining for STAT6 becomes a pedestal method to make pathologic diagnosis of SFTs more accurate.

## SYMPTOMS

More than 50% of the patients with SFT are asymptomatic, and the tumor is frequently an incidental finding on imaging. Symptoms, if any, are more frequently associated with larger/central, and generally malignant, tumors. SFT may rarely present with systemic symptoms (hypoglycemia, hypertrophic osteoarthropathy), or non-speciﬁc symptoms (fever, weight loss, fatigue) (12).

Several series have demonstrated that chronic cough, chest pain and dyspnea were the most common complaints. Chest pain occurs more commonly in patients with tumor arising parietal pleura or invasion of parietal pleura or thoracic wall (2,12).

Hypertrophic pulmonary osteoarthropathy (HPO) (also known as, Bamberger-Marie syndrome or Osteoarthropathia hypertrophicans) symptom complex occurs in many thoracic disease processes. It has been described in as many as 20% of the SFT cases (9), particularly in larger tumors (e.g. >7 cm) (13). HPO can resolve post-surgery (14). Digital clubbing has been reported in all improve within 2-5 months (median 4 months) of surgery, but may reappear with tumor recurrence (14).

Hypoglycemia, referred to as the Doege-Potter syndrome, apparently results from tumoral production of insulin-like growth factor II, and is reported to occur in a severe form in 2-4% of patients with (often large) SFT (9,15).

## RADIOLOGY

**Figure 1:**
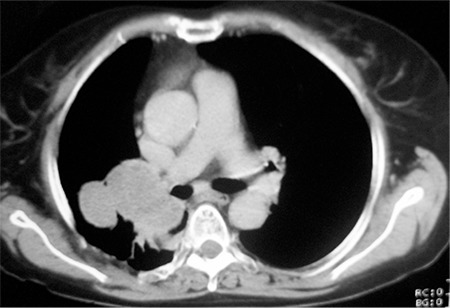
CT scan of the chest showing a lobulated mass with heterogeneous enhancement, compressing the mediastinum, right main bronchus and right pulmonary artery.

**Figure 2a, 2b:**
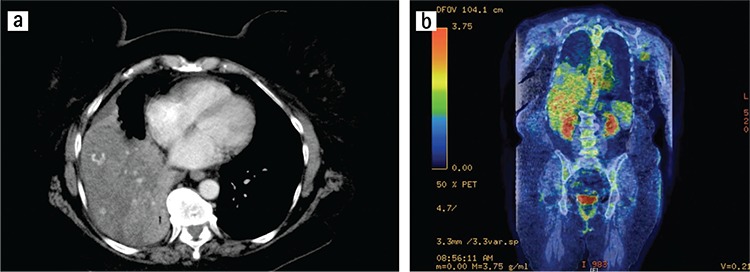
Axial CT and coronal PET-CT slices showing a huge malignant solitary fibrous tumor mass in the lower lobe of right lung.

**Figure 3:**
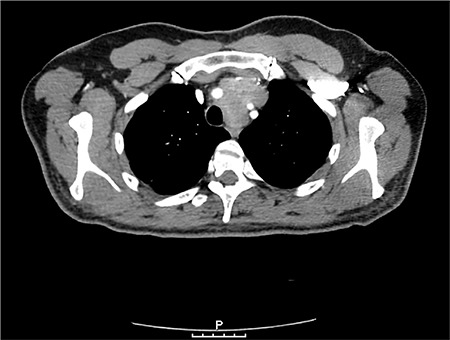
Axial CT slice of a solitary fibrous tumor located in anterior mediastinum.

**Figure 4a, 4b:**
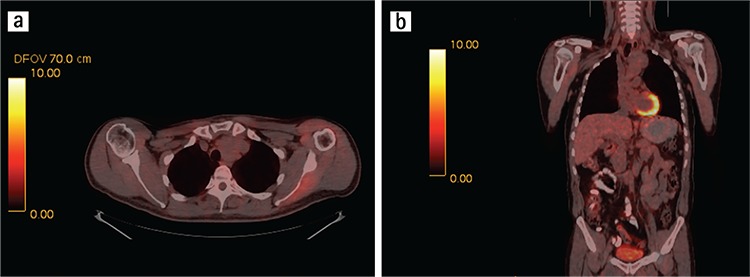
Axial and coronal PET-CT slices of a solitary fibrous tumor located in mediastinum which slightly compressed trachea.

SFT typically appear as masses abutting the pleura; most often arising from the lung visceral pleura (≈80% of cases) (13), but occasionally with apparent origins from the mediastinal (≈1-8% of cases) or parietal pleura (2,12,13,16,17). Given the close proximity of the various pleural surfaces to each other, the precise origin/extent of these lesions can be uncertain (Figure 1,2,3,4). The differential diagnosis typically includes mesothelioma, soft tissue sarcomas, and metastatic disease to the pleura (2,12).

### Chest Radiography

On plain chest radiographs, SFTs appear as a homogeneous round mass, with smooth and well-defined margins. Pedunculated tumors may change the radiographic silhouette during breathing and decubital positioning. Usually the mass forms obtuse angles with the pleural surface, but in the case of large masses the angle may be acute. Pleural effusions are not typically seen and destruction of underlying bone occurs rarely (18).

### Computed Tomography

Computed tomography (CT) scan is generally considered the optimal procedure to determine the size and location of the tumor, and is essential for surgical planning. CT attenuation depends on collagen content in these lesions. Thus, hyperdense lesions are typically collagen-rich, and low-attenuation regions represent necrosis or areas of myxoid or cystic degeneration (2,12,19). See Figure 1, 2, and 3 for examples.

### Magnetic Resonance Imaging

Magnetic resonance imaging (MRI) may be useful to evaluate SFT cases due to its excellent soft tissue resolution. MRI features of SFTs reflect the histologic findings and amount of fibrous tissue, necrosis, and hemorrhage. SFTs are typically characterized by low signal intensity on most MRI sequences, that is explained by the high collagen content within the tumor’s stroma and that may be helpful in making a preoperative differential diagnosis of a pleural-based mass. Thoracic SFTs typically have intermediate signal intensity on T1WI and heterogeneous low signal intensity on T2WI. Hyper intensity on T2WI is related to increased cellularity, edema, cystic degeneration, and hemorrhage. After gadolinium administration a marked heterogeneous enhancement is observed (2,20).

### Positron Emission Tomography

Fluoro-deoxy-glucose positron emission tomography (FDG PET) imaging can be helpful in differentiating more malignant conditions (e.g. lung cancers, pleural metastases, that are metabolically active on FDG PET) from SFT (that are largely indolent and not hypermetabolic on FDG PET). Large SFTs with increased FDG PET activity likely have a higher probability for malignancy, and in this setting the FDG PET might help to delineate the tumor extent local/regionally and distantly (example Figure 2 and 4) (21). Table 1 summarizes the general features of thoracic SFTs.

### Response Evaluation

The Response Evaluation Criteria in Solid Tumors (RECIST) is a commonly used system to evaluate clinical treatment response based on changes in tumor size. Since some new targeted therapies, may cause tumor necrosis (with a decrease in tumor density but without a marked decrease in tumor size), the RECIST approach may not be optimal in this setting. Modified versions of the RECIST approach have been suggested that consider tumor size and imaging characteristics (e.g. Choi, or Positron Emission Tomography Response Criteria in Solid Tumors) and may be useful in evaluating response to treatment in patients with SFT (22,23).

## BIOPSY

Since these tumor arise from the pleural space, without direct contact with the airspaces, sputum cytology is usually not helpful. In the occasional case with a pleural effusion, cytology may show suspicious cells, but often is not diagnostic (24,25). Given the relative low cellularity of these lesions, making a histologic diagnosis can be challenging even when the gross lesion is directly sampled. For example, percutaneous transthoracic aspiration may be unsuccessful in distinguishing between malignant and benign disease, due to insufficient tumor sampling (26). Sung et al. (27) reported that they were able to obtain a definite preoperative diagnosis of SFT with fine needle aspiration biopsy in 43% of cases were. Tru-cut neddle biopsy has been associated with a higher yield of 100% (5 to 5) by Weynand et al. (28). SFTs do not appear to have a high potential for seeding. However, there is a theoretical risk of tumor seeding along the biopsy tract following transthoracic procedures, and indeed there is at least one such case report for SFT (29). Video-assisted thoracoscopic biopsy may be considered a more optimal approach to achieve a definitive diagnosis. Often, a definitive diagnosis is not obtained until review of the resected specimen (2,12).

## TREATMENT

### Surgery

Surgery of SFTs consists of an en bloc resection of the tumor surrounded by a margin of healthy tissue. Relatively small tumors can be resected via video assisted thoracoscopic surgery, whereas large tumors may require thoracotomy (7). Caution should be used to avoid contact between the tumor and the thoracoscopic sites, as seeding at the port sites has been reported (30).

Complete surgical resection is considered an important prognostic factor and surgical margins of 1 to 2 cm into healthy lung parenchyma from the tumor is recommended (13). Whereas pedunculated tumors can be safely resected with a wedge resection of the lung, large sessile tumors can be difficult to resect because of extensive adhesions, and may occasionally require a lobectomy or a pneumonectomy in order to achieve complete resection (31). Resection of the adjacent chest wall is done if there is gross involvement/adherence apparent at the time of surgery. In ≈ ≤3% of patients, the tumors are reported to be “inverted” and grow inside the lung parenchyma (32). These tumors often require a lobectomy (8,9).

For localized recurrence of SFT, surgery may again be considered the optimal treatment. For patients with metastatic or technically inoperable SFTs, surgery may still play a role for palliation (e.g. relief of mass-related symptoms).

### Radiotherapy

**Adjuvant radiotherapy:** The available literature is inconclusive with regard to the utility of postoperative radiotherapy (RT) for SFT treatment. Extrapolation of data from patients with soft tissue sarcomas might be reasonable. In patients with soft tissue sarcomas of the extremities, RT has been shown to decrease local recurrence (LR) rate following surgery (33). There are a few published retrospective series addressing adjuvant RT of thoracic soft tissue sarcomas (34,35). For example, Duranti et al. (34), described their experience treating 337 patients with thoracic localized soft tissue sarcoma. The mean patient age was 50 years and median tumor size was 8 cm. Location was soft tissue and chest wall in 85.5%, mediastinum 9.5%, and pleura 5%. The majority (51%) received adjuvant RT, and 41% received postoperative chemotherapy. With a median follow-up of 4.7 years, the 5-year overall rate of LR rate was 14%. Radiation therapy was associated with better local control on both univariate and multivariate analysis (34).

In an analysis of 603 SFT cases (of 35% in thoracic localization) in the Surveillance, Epidemiology, and End Results database from 2000 to 2009, overall survival was similar for patients treated with surgery plus adjuvant RT compared to surgery alone. Given the nature of such population data, the patients in the different treatment groups were likely not similar. Presumably, the patients who received both RT and surgery had “worse tumors” (e.g. more likely close/positive margins) than those treated with surgery alone. Thus, the observation that the overall survivals are similar in the two treatment groups might suggest some utility to the RT. However, this is speculative. Nevertheless, this conclusion would be consistent with what we know from soft tissue sarcomas (36).

Krengli et al. (37) summarized treatment results 102 patients with SFT of which 23% had thoracic disease. Local control was higher in patients who received surgery plus postoperative RT vs. surgery alone.

RT has traditionally been used in patients with malignant SFT, narrow margins, large tumor size, or apparently-fast growing tumors. Histologically benign and completely resected tumors appear to have good outcomes irregardless of tumor size (31). However, tumors larger than 10 cm with malignant characteristics have been associated with a higher rate of disease recurrence. For example, de Perrot et al. (31) reported a recurrence rate maybe as high as 63% as for sessile malignant SFTs. In this patient group, postoperative RT should be considered even after complete resection.

Anecdotal reports describe long-term survival in those undergoing postoperative RT in the setting of incomplete resection. For example, Suter at al. (26) published a case report of a patient with over 20-year disease free survival after subtotal resection followed by RT. It might be prudent to consider post-operative RT in settings were a tumor recurrence might be expected to cause significant morbidity and/or not be readily amenable to re-resection.

A summary of reports using RT for SFT is provided in Table 2. Overall, postoperative RT likely can improve local control probability; especially in patients with a relatively high risk of local failure (e.g. malignant histology and/or large tumor size, surgical margin positive disease).

**Neoadjuvant radiotherapy:** For soft tissue sarcomas, preoperative RT is often used sterilize the anticipated resection margins and improve local control. The potential theoretical advantages of preoperative RT include reduced potential for tumor seeding during surgery, better tumor oxygenation leading to improved radiosensitivity of the intact tumor, reducing the risk of close or positive margins (38), and smaller RT fields (vs. post-operative RT). However, the use of pre-operative RT for SFTs is often limited by the difficulty of obtaining a precise preoperative diagnosis even with an open biopsy, and the unclear utility of this approach.

**Curative/Palliative radiotherapy:** In those with unresectable or metastatic disease, RT may provide a palliative option symptomatic local disease (e.g. pain or airway compression).

**Radiosensitivity and radiotherapy dose:** Soft tissue sarcomas have often been erroneously labelled as “radio resistant”. This might be due to lack of clinically evident tumor shrinkage during RT. However, this lack of shrinkage might be reflective of the low cellularity, extensive necrosis, and dense fibrotic connective tissues, within many sarcomas, rather than the lack of RT-induced cell death. Indeed, clinical data suggest clinical efficacy as described.

Clonagenic survival assays of human soft tissue sarcoma lines reveal no consistent evidence of intrinsic resistance to radiation. The surviving fraction at 2 Gy dose (SF2) has been accepted as a clinically relevant measure of radiation sensitivity. Several studies note SF2 values in the range of 0.124-0.39 (average 0.24), similar (and *perhaps even lower; i.e. more sensitive*) to those seen in carcinomas (generally considered ‘radiosensitive’ base on changes in tumor size seen in the clinic) (38,39,40,41).

In addition, the radiation doses that effectively reduce local failure following surgery for adenocarcinaoma of the breast, or squamous cell carcinoma of the cervix or head and neck, are similarly able to reduce local failure when combined with surgery for soft tissue sarcoma (42,43).

RT alone may provide local control in many patients (e.g. ≈30-60%) with sarcoma who elect to not undergo recommended surgery or are deemed unsuitable for surgery (42). Similarly, RT alone (to ≈ 60-70 Gy) leads to responses in up to 50% of unresectable lesions (36,37). For certain radiosensitive histological subtypes (perhaps myxoid liposarcoma), pre-operative RT may be particularly advantageous, as significant tumor shrinkage can be observed (42,43,44).

These observations suggest that the alleged “radioresistance” of sarcomas may be without basis.

For SFT, there are multiple case reports documenting clinical responses. For example, Kawamura et al. (45) reported a patient with a pelvic SFT with multiple lung metastases, who had signiﬁcant response to 50 Gy pelvic RT. Saynak et al. (46) reported a significant response with 60 Gy thoracic RT in 30 fractions in a patient with recurrent malignant SFT. Liu et al. (47) also reported a case where RT alone achieved a 44% decrease in tumor size, without evidence of progression at 10 months follow-up post-RT.

The pace of tumor shrinkage following RT is related to the underlying tumor kinetics. Tumors that grow rapidly tend to shrink rapidly, and vice versa. Given the relatively-indolent clinical course of SFT, one might expect RT-associated tumor shrinkage to be delayed (i.e. more than a few weeks/months). Indeed, in the four case reports of tumor response following RT, the maximum response was seen at ≈3-10 months post-RT (45,46,47,48). Some curative/palliative RT experiences are summarized in Table 2.

The optimal pre/post-operative RT doses in patients with SFT is unclear, and extrapolation from other tumor sites is reasonable. The literature reported post-operative doses usually range from 45-60 Gy (37,49,50,51). In cases where the perceived risk of LR is higher (e.g. close margins), it is reasonable to more-routinely consider using higher doses (eg, 54-60 Gy or even 60-66 Gy for positive margins) (43,51). In these settings, we advise that the RT fields be made as tight as possible to minimize the incidental doses to normal tissues. Newer technologies such as IMRT/IGRT techniques can be used to more accurately deliver RT. For soft tissue sarcomas, the standard regimen for pre-operative RT is 45-50 Gy, in 1.8-2 Gy fractions, followed by surgery approximately 6 weeks following completion of RT, and this same approach might be warranted if pre-operative RT were to be used for SFT (43).

## SYSTEMIC TREATMENT

Data regarding the effectiveness of systemic therapy for SFT is limited (Tables 3, 4). In general, the reported response rates are low. However, results are perhaps somewhat more encouraging with Dacarbazine, or some of the newer targeted agents (52,53,54,55,56,57,58,59).

## PROGNOSIS

The prognosis for SFT is variable. Broadly speaking, worse prognosis has been associated with large size, malignant histology and pathologic characteristics (7,9,14,27,60,61,62,63,64,65,66,67,68,69,70,71,72) (See Table 5).

Tapias et al. (68) proposed a scoring system which combines common clinical and histological features able to predict the recurrence after (radical) surgical resection for thoracic SFTs. Their criteria were pleural origin (visceral/intrapulmonary or parietal), morphology (pedunculated or sessile), size (<10 cm or ≥10 cm), hypercellularity, necrosis or hemorrhage and mitosis number (<4 or ≥4). They assigned one point for each unfavorable variable. In their analysis, a sum of <3 points was associated with a low risk of recurrence (e.g. 3.5% at 15 years) compared with recurrence rate of 28% in patients with ≥3 points. They found that the presence of a pleural effusion, as well as a symptomatic presentation, and a Ki67 proliferation index >10% were associated with SFT recurrence on univariate analysis. Unfortunately, these variables could not be included in this regression models given the paucity of events and the small sample size (67). This new risk stratification model will need to be validated in other cohort studies.

The 10-year overall survival rate varies between 54 and 89% among published series (7,9,14,27,60,61,62,63,64,65,66,67). Metastatic disease is often only appreciated 10-20 years after initial diagnosis, and bone, lung, and liver appear to be common sites of metastatic disease. Thoracic metastases are discovered in 0-36% and extra-thoracic metastases are found in 0-19% of patients with pleural based SFT (2,12). Unfortunately, most patients with recurrent disease survive less than 5 years (7). Despite systemic therapy, patients with metastatic disease have a poor prognosis with median survival ranging from 22 to 46 months (52,54).

Published large series demonstrated that one of the most important prognostic factor is whether the tumor can be completely excised initially (10). Unfortunately, even completely removed lesions may recur. LRs may occur even after 5 years after surgical operation. Recurrence after complete resection of a benign SFT can be either benign or malignant. Most patients who have recurrence in the setting of malignant disease survive less than 5 years (2,7,12).

In conclusion, 1. SFT is a fibroblastic mesenchymal neoplasm that arises in both thoracic and extrathoracic sites. Thoracic forms are generally asymptomatic at the time of diagnosis, and the radiographic features are a well-circumscribed, peripheral mass that abuts the pleural surface, frequently attached by a pedicle. Pediculated and sessile SFTs of thoracic sites commonly grow into the pleural cavity; however, some rare cases grow into the lung parenchyma.

2. Occasionally larger lesions may cause local symptoms (e.g. cough, chest pain and dyspnea).

3. Approximately 10% to 20% of SFTs are histologically classified as malignant (e.g. with increased mitoses, necrosis, atypia, and hypercellularity), and these may be hyperactive on PET.

4. Histological and morphological characteristics are often predictive of outcome. Malignant SFTs have a higher recurrence rate and mortality rate (up to ≈30% in some series).

5. Surgical excision has been the standard treatment option for both benign and malignant SFTs, and is both diagnostic and therapeutic. The role of adjuvant RT or chemotherapy is unclear. Nevertheless, RT can be considered in cases of SFTs with malignant features, especially in the presence of incomplete excision.

6. Some newer systemic agents (e.g. sunitinib, sorafenib and bevacizumab) may be helpful for patients with unresectable or metastatic tumors. Post-treatment imaging can show tumor devascularization detectable on scans without much size change in tumor volumes, and thus the classic RECIST criteria may not be optimal in quantifying response. Modified RECIST crieteria might be more appropriate.

7. Reports suggest that RT can provide an apparent responsive effect on SFTs in similar fashion to other soft tissue sarcomas. Inoperable/recurrent localized disease may also be treated with RT.

8. SFTs can have very late recurrences (>15 years), and this long-term clinical follow-up is recommended.

## Figures and Tables

**Table 1:**
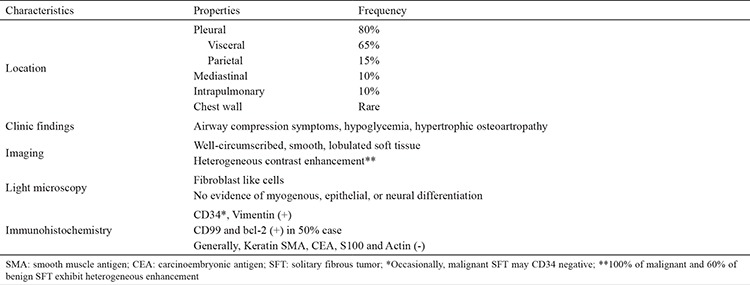
Characteristics of SFTs of the thorax

**Table 2:**
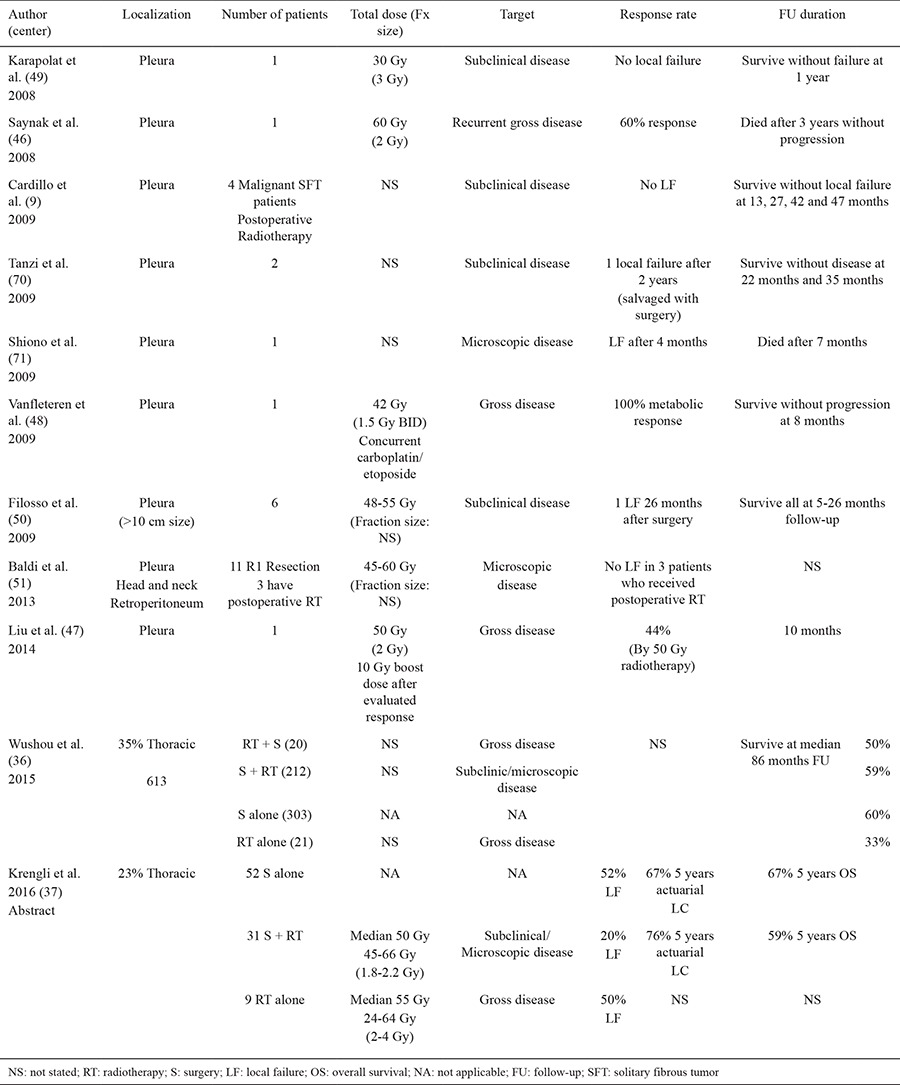
Reports of radiation therapy for SFT

**Table 3:**
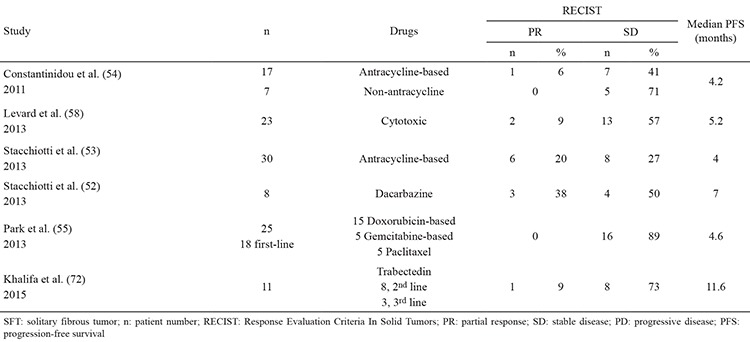
Reports of conventional chemotherapy for SFT

**Table 4:**
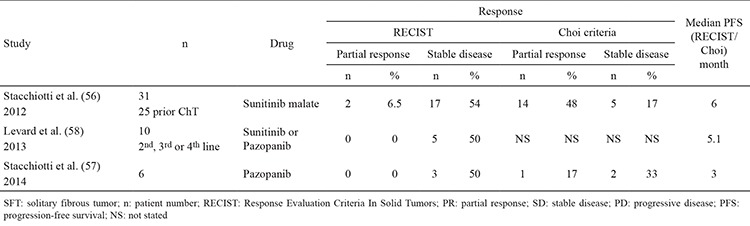
Reports of targeted agents for SFT

**Table 5:**
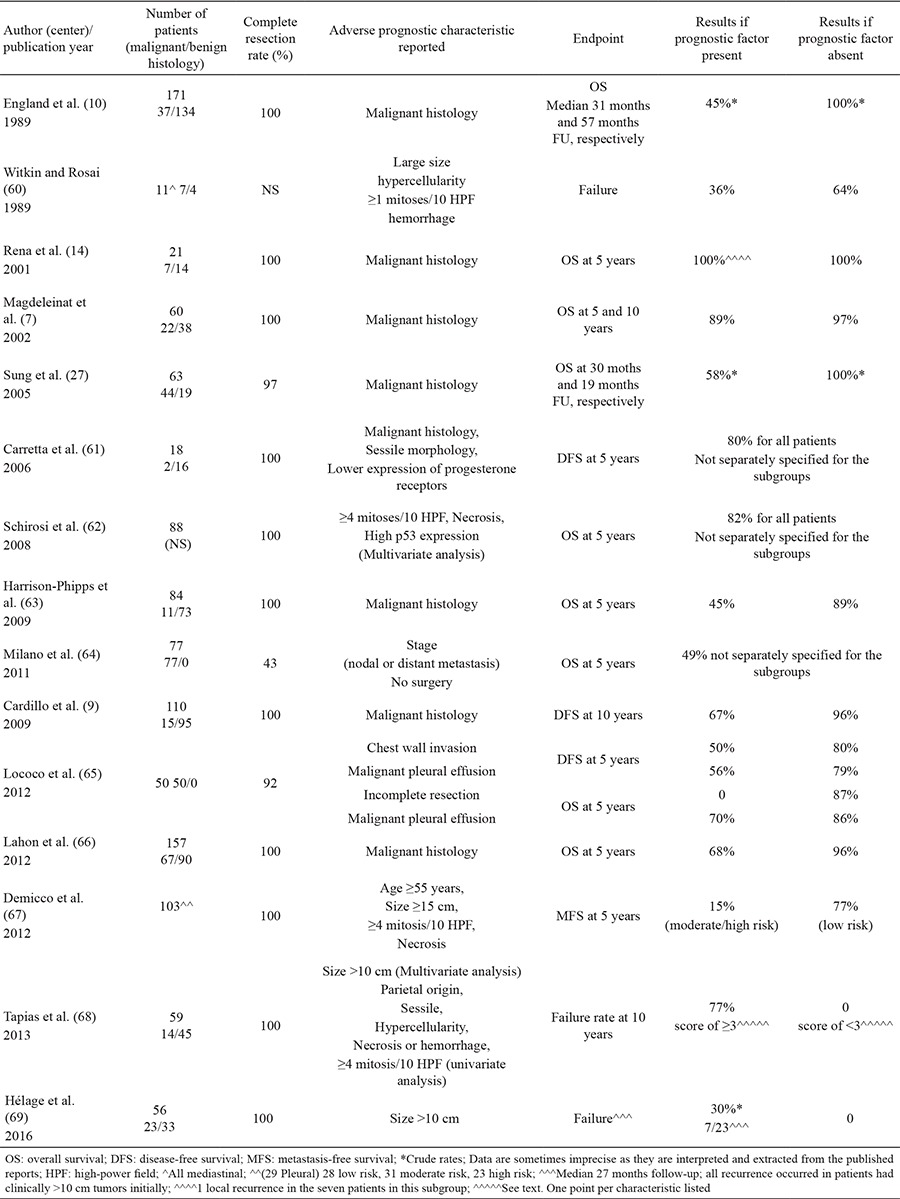
Tumor characteristics associated with a poorer prognosis in surgical series published after malignancy criteria defined (1989)
